# Species breadth is a preparedness metric: lessons from the 2026 Bundibugyo virus disease outbreak

**DOI:** 10.1080/22221751.2026.2744490

**Published:** 2026-10-05

**Authors:** Ayan Mohamed Hassan, Abdullahi Abdi Yusuf, Adan Mohamed Hirsi, Ilyas Abdullahi Khalif, Ayaan Axmed Maxamed, Deka Ahmed Abdi

**Affiliations:** a Faculty of Medicine and Surgery, Somali National University, Mogadishu, Somalia; b Banadir Research Consultancy & Evaluation Center, Mogadishu, Somalia

**Keywords:** Bundibugyo virus, Ebola virus disease, early detection, diagnostics, medical countermeasures, outbreak preparedness

## Abstract

Recent outbreaks of non-Zaire orthoebolaviruses have exposed a persistent weakness in outbreak preparedness: diagnostic, vaccine, therapeutic, regulatory, and research systems remain disproportionately optimized for Ebola virus (EBOV). The 2026 Bundibugyo virus disease outbreak in the Democratic Republic of the Congo and Uganda illustrates the consequences of this species restriction. Initial testing with an EBOV-focused assay was negative, whereas broader molecular testing and genomic sequencing subsequently confirmed Bundibugyo virus, highlighting a gap between clinical suspicion and species coverage. This Commentary proposes an auditable species-breadth profile across four preparedness domains: diagnostics, vaccines, therapeutics, and evidence generation. For a minimum audit covering EBOV, Sudan virus, and Bundibugyo virus, each domain can be scored from 0/3 to 3/3 using predefined criteria for deployable detection, countermeasure availability, access pathways, and the capacity to initiate rigorous research during an outbreak. Species-breadth assessments should be integrated with specimen referral, genomic sequencing, cross-border surveillance, responder protection, community engagement, and contingency financing. Preparedness should, therefore, be judged not only by the availability of tools for one Ebola virus species, but by the ability of surveillance, diagnostics, countermeasures, and research systems to respond rapidly to whichever pathogenic orthoebolavirus emerges next.

The rapid, unchecked growth of the 2026 Bundibugyo virus disease outbreak in the Democratic Republic of the Congo (DRC) and Uganda should not be interpreted merely as a consequence of delayed outbreak detection. It exposes a deeper limitation in Ebola preparedness: many diagnostics, vaccines, therapeutics, regulatory pathways, and deployment systems developed after 2014 were optimized primarily for Ebola virus (EBOV; Zaire ebolavirus) and were not translated with equal urgency to other pathogenic orthoebolaviruses [1,2]. Recent reviews have already called for integrated, species-inclusive preparedness [3]. The contribution of this Commentary is therefore not to restate that principle, but to make species breadth measurable.

We propose an auditable species-breadth profile across four domains: diagnostics, vaccines, therapeutics, and evidence generation. For each domain, readiness is the number of priority epidemic orthoebolaviruses meeting a predefined criterion divided by the number assessed. For a minimum audit of EBOV, Sudan virus (SUDV), and Bundibugyo virus (BDBV), the denominator is three, producing a domain-specific score from 0/3 to 3/3. Diagnostic breadth requires deployable first-line or reflex detection; vaccine and therapeutic breadth requires evidence-supported products and deployment pathways; evidence-generation breadth requires pre-reviewed protocols, trial networks, regulatory pathways, and access to investigational products. Scores should remain separate because strength in one domain can coexist with a critical gap in another.

Early detection remains fundamental because delayed recognition is associated with larger, longer outbreaks [4]. In 2026, the first currently known suspected case developed symptoms on 24 April, WHO was alerted on 5 May, and initial testing of 20 samples with the standard Xpert Ebola assay was negative. After referral to the Institut National de Recherche Biomédicale, 13 blood samples were analyzed on 14 May and BDBV was confirmed on 15 May by broader PCR and genomic sequencing [5]. This sequence distinguishes the prolonged clinical-recognition delay from the shorter referral-to-confirmation interval. Xpert Ebola was developed for EBOV and targets the glycoprotein (GP) and nucleoprotein (NP) genes [6]; sequence divergence in BDBV can impair primer/probe recognition [1,6]. The negative results therefore reflect intended target coverage rather than assay failure and should not be treated as the sole explanation for delayed recognition. They do, however, expose a diagnostic blind spot, consistent with recent calls for reliable BDBV rapid testing [7,8].

Broad-range molecular assays show that wider coverage is technically feasible, although breadth does not guarantee identical sensitivity. The RealStar Filovirus Screen detects EBOV, SUDV, BDBV, Taï Forest virus, Reston virus, and Marburg virus, but its EBOV-specific derivative performs better at low EBOV concentrations [9]. A pan-filovirus RT-qPCR showed 100% concordance with an EBOV-specific reference assay in 83 clinical samples, while analytical detection limits varied across species, including approximately 9 copies/reaction for EBOV, 65 for BDBV, and 1151 for SUDV [10]. Preparedness assessments should therefore report both species coverage and species-specific analytical and clinical performance, with explicit reflex pathways for clinically compatible cases that test negative on species-specific platforms.

The countermeasure landscape shows a parallel asymmetry. Post-2014 investment accelerated EBOV vaccines and therapeutics [11], but no licensed BDBV vaccine or specific therapeutic was available for routine deployment in 2026 [1,5]. Yet the need to evaluate a candidate during an outbreak is not itself a preparedness failure. PARTNERS was a pre-established WHO-sponsored adaptive platform with a core filovirus therapeutics protocol published before the outbreak [12], and it was rapidly activated to evaluate MBP134 and remdesivir [13]. The response therefore demonstrates limited product readiness but comparatively strong evidence-generation readiness. For rare, episodic pathogens, preparedness must include both deployable products where evidence permits and the infrastructure to generate rigorous efficacy evidence quickly when it does not.

The 2022 Uganda SUDV outbreak demonstrates that this asymmetry is recurrent. RT-PCR confirmation was available, but no licensed SUDV vaccine or specific therapeutic existed, and candidate countermeasures required evaluation during the emergency [14]. SUDV caused 224 deaths in Uganda in 2000 and 55 confirmed deaths in the 2022 outbreak [14]. In 2026, BDBV added a frontline assay-coverage gap to a similar product-readiness gap. Historical experience also shows that non-EBOV species are not theoretical edge cases: BDBV caused recognized outbreaks in Uganda in 2007–2008 and the DRC in 2012 [3], while Taï Forest virus caused a documented human infection in Côte d’Ivoire in 1994, with recovery and no identified secondary transmission [15]. TAFV therefore remains relevant to broad surveillance, while the minimum 3/3 audit focuses on the three species with repeated epidemic human transmission.

These technical gaps are compounded by insecurity, population movement, humanitarian need, mistrust, and risks to responders, all of which can weaken surveillance, contact tracing, safe burial, isolation, and continuity of care [1,2,5]. Species breadth should therefore be assessed within operational systems that integrate laboratory readiness with community engagement, responder protection, cross-border coordination, and clinical-care capacity.

Four measurable priorities follow. First, every designated Ebola-testing network should document access, at first-line or immediate-reflex level, to molecular detection of EBOV, SUDV, and BDBV (3/3), with automatic broader orthoebolavirus testing after a negative species-specific result. Second, vaccine and therapeutic breadth should be reported separately by species; where no approved deployable product exists, preparedness should document at least one candidate with pre-outbreak human safety data, a defined regulatory-access pathway, and reserved outbreak supply. Third, evidence-generation readiness should require a current master protocol, pre-reviewed country/site documents, named trial sites, and product-access agreements, reviewed annually and tested through simulation. Fourth, each designated high-risk border district should have a mapped specimen-referral route, access to genomic sequencing, a cross-border data-sharing agreement, and a documented contingency-financing mechanism with named activation authority, verified through at least one annual cross-border exercise.

The evidence from an ongoing epidemic remains incomplete, and final severity, transmission dynamics, and product effectiveness require caution. Nevertheless, the 2026 BDBV outbreak already shows that preparedness can be strong in some domains and species-restricted in others. Species breadth offers a simple way to make those asymmetries visible. Readiness should not be judged by whether effective tools exist for one ebolavirus, but by whether surveillance, diagnostics, countermeasures, regulation, and research systems can respond rapidly regardless of which pathogenic orthoebolavirus emerges next.

## Data Availability

No datasets were generated or analyzed during this study because this Commentary is based on narrative synthesis and interpretation of published literature.
